# Exploring early maladaptive schemas in healthy eating and orthorexia nervosa tendencies: a structural equation modeling approach

**DOI:** 10.1186/s40337-026-01572-3

**Published:** 2026-03-27

**Authors:** Alexandra Fodor, Balázs András Varga, Adrien Rigó

**Affiliations:** 1 Doctoral School of Psychology, ELTE Eötvös Loránd University, Budapest, Hungary; 2 Institute of Psychology, ELTE Eötvös Loránd University, Budapest, Hungary

**Keywords:** Orthorexia nervosa, Healthy orthorexia, Early maladaptive schemas

## Abstract

**Purpose:**

This study aimed to examine the relationship and differences between healthy eating, orthorexia nervosa tendencies and early maladaptive schemas.

**Methods:**

A cross-sectional online survey was conducted on a sample of Hungarian adults. One thousand twelve participants completed measures assessing healthy orthorexia, orthorexia nervosa tendencies, perceived stress, and early maladaptive schemas. Structural equation modeling was used to determine the relationships among the variables.

**Results:**

Results indicated significant associations between orthorexia nervosa tendencies and specific early maladaptive schemas, particularly unrelenting standards and defectiveness/shame schemas. Additionally, age, perceived stress, and BMI were found to be associated with orthorexia nervosa tendencies and healthy orthorexia.

**Conclusions:**

The findings suggest that early maladaptive schemas, especially those related to perfectionism and low self-esteem, may contribute to the development and persistence of high orthorexia nervosa tendencies. More research is necessary to examine these relationships and their potential therapeutic implications.

**Level of evidence:**

Level V, descriptive cross-sectional study.

## Abstract (plain language summary)

We all hold deep-rooted beliefs about ourselves that influence our thoughts, feelings and behaviors. One type of belief, called „early maladaptive schemas,” can develop during childhood if basic emotional needs are not met. Since previous research has shown that these schemas contribute to the development of anorexia and bulimia nervosa, we examined whether they also play a role in orthorexia nervosa tendencies. Although orthorexia nervosa is not formally recognized as a distinct diagnosis within current classification systems, its characteristic symptoms, such as strict adherence to extreme dietary rules, eating only foods considered „healthy”, and feeling of shame following dietary violations, can lead to significant distress and impairments in affected individuals. Our findings indicate that individuals with orthorexia nervosa tendencies are more likely to display stronger activation of the unrelenting standars schema, characterized by the pursuit of excessively high personal standards, as well as the defectiveness/shame schema, which reflects pervasive beliefs of inadequacy or unworthiness. The unrelenting standards schema was also linked to healthy eating habits, while the defectiveness/shame schema was only associated with orthorexia nervosa tendencies, suggesting a possible role in the disorder. We also observed some unexpected results: healthy eating habits were positively related to perceived stress, whereas orthorexia nervosa tendencies were negatively related to it. This pattern may reflect the challenge in determining when healthy eating transitions into maladaptive behavior, and may also suggest that individuals with orthorexia nervosa tendencies might experience positive emotions as a result of strict routines and control.

## Background

Orthorexia nervosa (ON) typically presents as an excessive preoccupation with healthy eating, often motivated by a desire to improve health, frequently following a diagnosis of a chronic illness or as part of recovery from a previous eating disorder [1–3].

What initially begins as an attempt to improve health can escalate into restrictive eating, compulsive behaviors and an obsessive focus on consuming only foods perceived as healthy [1, 4]. ON is not currently included in any official diagnostic classification system [5, 6], although there is a proposed (not official) diagnostic criteria based on recent guidelines; therefore, when referring to orthorexia nervosa in this article, we are discussing tendencies, predispositions, or symptoms of orthorexia nervosa, not a formally recognized or diagnosable mental disorder. (For symptoms based on the recently proposed diagnostic criteria, see Table 1.)

**Table 1:** Symptoms of Orthorexia nervosa tendencies

1.	Individuals with higher orthorexia nervosa tendency follow rigid, self-imposed rules, eating only what they view as “pure”, or “healthy”, while avoiding “unhealthy” or “toxic” foods [5, 17, 18].
2.	ON often becomes central to their identity, provides a sense of control, with excessive time spent planning, preparing or eating their meals [2, 5].
3.	They perceive their behavior as ego-syntonic, and also experience moral superiority [2, 41].
4.	Their diet typically becomes increasingly restrictive, causing emotional distress when they eat food they label as “unhealthy”, and food transgressions are often followed by guilt, worry, anxiety and self-punitive behavior, leading to more restrictions in their diet [2, 4, 5, 19].
5.	Individuals with ON tendencies also often experience cognitive impairments, such as problems with attention or concentration [5].
6.	Nutritional imbalances from the strict diet can result in health issues, including malnutrition, low weight, hormonal or gastrointestinal problems, and ON tendencies tend to impact mental, social and even daily functioning [2, 5, 17, 18, 41, 55].

Distinguishing ON tendencies from adaptive forms of healthy eating is essential for understanding the condition. One perspective questions the idea that healthy eating exists on a single continuum, ranging from complete disinterest to orthorexia, with healthy eating in between [7]. The two-dimensional model of the Teruel Orthorexia Scale provides a more detailed framework for differentiating adaptive healthy eating from ON tendencies. In this model, Healthy Orthorexia (HeOr) reflects an interest in and commitment to healthy eating without associated impairments. Individuals with high HeOr tendencies perceive healthy eating as a positive lifestyle choice and dedicate energy and time to it. In contrast, the Orthorexia Nervosa (ON) factor captures emotional distress, self-punishment, guilt, and social isolation related to eating habits [4, 7]. ON is more strongly linked to psychological distress, restrictive eating, obsessive-compulsive symptoms, perfectionism, and low self-esteem, whereas HeOr shows weaker or negative associations with these variables [7–10]. Additionally, individuals with high ON scores may use healthy eating as a way to regulate their emotions [4, 11–13].

Early Maladaptive Schemas (EMS) are pervasive, deeply rooted patterns of emotions, bodily sensations, and cognitions. According to Young, EMS develop when basic childhood emotional needs remain chronically unmet [14–16]. EMS arise mainly within one’s relationship with oneself and others, and their dysfunctional nature contributes to recurring emotional and behavioral patterns and difficulties experienced in adulthood [14–16]. Because EMS are ego-syntonic and can become part of one’s identity, they are often difficult to recognize. Individuals usually only see the surface symptoms and may not realize that their recurring issues stem from the same underlying schema [14]. For example, someone with an unrelenting standards schema might be highly perfectionistic in work, relationships, and their expectations of others. Although they may perceive their challenges as missed deadlines, overcommitment, exhaustion, or strained relationships, they often fail to see how their perfectionism and overly self-critical and critical attitude toward others fuel these problems. While the importance of early maladaptive schemas has been thoroughly examined in the field of eating disorders, no similar research has been conducted for ON to date. Given this gap, we can draw on literature related to other eating disorders that share many similarities with ON, as well as from literature related to obsessive-compulsive disorders [17].

One core characteristic of ON tendencies is strict adherence to rigid dietary rules and a pervasive form of perfectionism. Both perfectionism and rigidity are commonly associated with the unrelenting standards schema [14]. Individuals with a strong unrelenting standards schema tend to follow rules rigidly, display perfectionism, and experience difficulties in relaxing or maintaining their health and interpersonal relationships [14]. These patterns are frequently observed in individuals with high ON tendencies [5, 17–19]. Multiple studies have demonstrated increased activation of the unrelenting standards schema in eating disorders, obsessive-compulsive disorder (OCD), and obsessive-compulsive personality disorder (OCPD) [20–22].

Another characteristic of ON tendencies is that self-esteem is often closely tied to adherence to dietary rules [5, 17, 23]. Violating these rules may lead to self-devaluation and self-punishment [5, 17, 23]. The association between low self-esteem and eating disorders is also well established [24–26]. From a schema therapy perspective, low self-esteem can be conceptualized as the defectiveness/shame schema, which has been shown to be more strongly activated in individuals with eating disorders, OCD, and OCPD compared to healthy populations [21, 22, 27–29]. Furthermore, low self-esteem and high levels of perfectionism (or the co-occurrence of the defectiveness/shame and unrelenting standards schemas) may increase vulnerability to developing ON tendencies [23, 30–33].

The literature has long emphasized the role of attachment in the development of eating disorders, with research indicating stronger activation of attachment-related EMS in individuals with eating disorders, OCD, and OCPD [21, 22, 27, 28, 34–38]. Several studies have also linked higher levels of ON tendencies to insecure attachment [25, 39, 40], a pattern that may be further reinforced by the tendency of individuals with ON to make adherence to dietary rules the central focus of their lives, often at the expense of their relationships [5, 19, 39]. In line with these interpersonal patterns, schemas within the Disconnection and Rejection domain (e.g., mistrust/abuse, emotional deprivation, abandonment/instability, defectiveness/shame, and social isolation) may also contribute to ON tendencies. (For a description of the early maladaptive schemas see Table 2).

**Table 2:** Description of early maladaptive schemas

Early maladaptive schema	Definition
Emotional deprivation	Anticipating, that others will fail to meet the individual’s normal need for emotional support. This may manifest as a lack of care (e.g. attention, emotional warmth), a lack of empathy (e.g. understanding, absence of mutual emotional sharing) or a lack of protection (e.g. absence of guidance, strength, and security) [14].
Abandonment/instability	A deep-rooted belief, that those from whom the individual seeks support or attachment, are unstable or unreliable. This includes the expectation that these significant others are incapable of providing long-term emotional support and attachment due to being emotionally unstable, unpredictable or untrustworthy, or that they will abandon the individual in favor of someone better or due to imminent death [14].
Social isolation	A deep-rooted belief, that the individual is completely isolated from the world, fundamentally different from others, and does not belong to or is not a part of any group or community [14].
Defectiveness/shame	A deep-rooted belief that the individual is fundamentally flawed, bad, undesirable, inferior or worthless in a significant way, and that others would be unable to love them if they truly knew them. This is often accompanied by constant self-comparison, insecurity, feelings of shame and sensitivity to criticism [14].
Mistrust/abuse	Anticipating, that others will harm, exploit, abuse, decieve, betray, lie to, manipulate or humiliate the individual. This belief is accompanied by the feeling that such actions are intentional or result from injustice or indifference [14].
Unrelenting standards	A deep-rooted belief, that the individual must meet extremely high, internalized standards for performance and behavior. Individuals with this belief often feel constant pressure, struggle to slow down, and may become excessively self-critical. They tend to adhere rigidly to rules, and „must” statements across various aspects of life, exhibit perfectionism and an excessive focus on details. As a consequence. They often experience significant impairments in pleasure, relaxation, health, self-worth and interpersonal relationships [14].

The aim of our study is to explore the relationships between ON tendencies and specific EMS, and to compare these relationships in individuals displaying ON tendencies versus those practicing healthy eating habits. At the time of our research, no prior studies had directly examined the link between ON tendencies and EMS. Therefore, we selected schemas that, based on existing literature, appeared theoretically relevant to both ON tendencies and healthy eating [16, 17, 22, 40]. Research highlights the prominent roles of rigidity and perfectionism in ON tendencies- traits also observed in OCD, OCPD, and AN [5, 17, 41]. Furthermore, the literature emphasizes the role of attachment issues in the development of AN, and several studies have demonstrated a relationship between insecure attachment and ON tendencies [31, 34, 38–40]. For this reason, we focused on maladaptive schemas related to attachment, and also included the unrelenting standards schema in our research.

Since our goal was to compare the activation of early maladaptive schemas in relation to both healthy eating and orthorexia nervosa tendencies, we aimed to choose a measurement tool that effectively distinguishes between the two. Therefore, we decided to use the Teruel Orthorexia Scale [7]. The present study aimed to determine whether early maladaptive schemas that have been shown to be more prevalent in disorders sharing characteristics similar to orthorexia nervosa are also elevated among individuals with ON tendencies. Specifically, the EMS assessed included Emotional Deprivation, Abandonment/Instability, Mistrust/Abuse, Social Isolation, Defectiveness/Shame, and Unrelenting Standards. Furthermore, considering the frequent association of ON tendencies with heightened levels of psychological distress and anxiety, we also examined the impact of these factors within our model. We hypothesized that the selected schemas would demonstrate stronger associations with ON tendencies compared to healthy eating tendencies. In particular, we anticipated that the defectiveness/shame and unrelenting standards schemas would exhibit the strongest associations with ON tendencies.

## Methods

### Procedure

The study was approved by the Ethics Committee of Eötvös Loránd University (Registration number: 2022/559). Participants were recruited via social media, and data were collected online using a Qualtrics survey battery accessible through an anonymous link. Recruitment took place over one academic semester in 2022. The questionnaire was shared on Facebook and Instagram, mainly through Hungarian psychoeducational pages focused on general mental health topics, as well as in Facebook groups centered on healthy eating. Participants were informed that the study involved completing a questionnaire assessing their eating habits and potential links between these habits and certain psychological traits. Participation was voluntary, and no compensation was offered. Inclusion riteria required participants to be at least 18 years old and to have no history of diagnosed mental or neurological disorders.

We excluded all participants who did not fully complete the questionnaire, as well as those with highly questionable response validity. After the screening process, 50.32% of the original data remained for analysis. To determine if the screening caused any bias compared to the original sample, we performed a logistic regression analysis using the available demographic variables. The regression model demonstrated a negligible effect size (Pseudo R2 = 1%), suggesting that the screening process did not cause significant bias in the sample. Only the level of education showed a statistically significant difference, though it was practically minimal. Individuals with higher education were about twice as likely to be retained in the final dataset compared to those with only primary education (*p* =.018).

Participants provided informed consent, completed demographic and dietary preference questions, and then filled out the Hungarian version of the Teruel Orthorexia Scale (TOS), the Perceived Stress Scale Short-Form, and the Young Schema Questionnaire-Short Form [7, 42, 43].

### Participants

Our study involved a total of 1012 participants, of whom 91.6% were women, while the remaining participants identified as men or other genders. The average age was 33 years. Data were collected from a Hungarian sample, but ethnicity was not assessed. More than 70% of participants were in a relationship. 63% had an university or college degree, and 33% completed high school. Most participants had an average (58.5%) or above-average (29.3%) standard of living. Based on self-reported height and weight, the average BMI was 24, with 65% falling within the healthy weight range (18.5 ≤ BMI ≤ 24.9). In addition, 45.6% of the participants followed a specific diet (See Table 3 for more detailed demographic information).

**Table 3:** Sociodemographic and other descriptive participant data

Variables	Mean ± SD
Age (years)	32.96 ± 11.11
BMI	23.27 ± 4.55
BMI ranges	
Underweight	73 (7.2)
Healthy weight range	659 (65.1)
Overweight	182 (18)
Obese	86 (8.5)
Gender	
Female	927 (91.6)
Male	81 (8)
Other	4 (0.4)
Marital status	n (%)
Single	299 (29.5)
In a relationship (not living together	139 (13.7)
In a relationship (living together)	574 (56.7)
Level of education	
Primary school	18 (1.8)
Vocational school	17 (1.7)
High school	337 (33.3)
University	640 (63.2)
Special diet	
Gluten-free	107 (10.6)
Lactose-free	124 (12.3)
Low-carb	112 (11.1)
Bio	10 (1)
Vegetarian	71 (7)
Vegan	109 (10.8)
No diet	551 (54.4)

*SD*, standard deviation; *BMI*, Body Mass index, *Bio diet*, Consuming only foods that are produced under natural conditions without the use of chemicals and without genetic modification, *Vegetarian*, Not consuming meat, *Vegan*, Not consuming animal-derived products

Regarding the distribution of Orthorexia Nervosa (ON) and Healthy Orthorexia (HeOr), most participants obtained scores in the middle range on the HeOr scale. Conversely, on the ON scale, the majority scored low, with only 40 participants scoring above 20 points, indicating a potential risk for orthorexia nervosa tendencies, even though there is currently no cutoff point for diagnosing ON.

### Instruments

#### Teruel Orthorexia Scale

The Teruel Orthorexia Scale contains 17 items, and requires participant responses on a 4-point Likert-scale (0 = Completely disagree, 3 = Completely agree). The TOS examines orthorexia in two dimensions: Healthy Orthorexia (HeOr) and Orthorexia Nervosa (ON). The two subscales are computed by summing the related items. For the purposes of the present study, the scale was translated to Hungarian, re-translated to English, and a bilingual psychologist conducted the final re-translation into Hungarian. In previous studies, Cronbach alpha values ranged between 0.85 and 0.9 for HeOr [7, 9, 44–46], while for the ON scale, values ranged between 0.81 and 0.91 [7, 9, 44–46]. Previous studies have found a moderate relationship between HeOr and ON scales, with values ranging between 0.4 and 0.6 [7, 9, 44, 46].

Since the two-factor adaptation of the TOS scale had not yet been performed in the target population, we first examined whether the two-factor CFA structured provided an adequate fit in the present sample.

All fit indicators were good in the study sample except for the sample size sensitive chi-square test (χ^2^(118) = 746.572, *p*<.001, CFI=0.973, TLI=0.969, RMSEA=0.073 CI90%=0.068–0.078.068.078). Estimation was performed using Diagonally Weighted Least Squares (DWLS) method due to the robustness of the procedure, given that each item was measured on 4 point scale and this approach is suitable for analyzing ordinal data. The reliability estimates in the sample were good for both the HeOr factor (α = 0.847, ω = 0.850) and the ON factor (α = 0.842, ω = 0.849).

#### Percieved Stress Scale-Short Form

The questionnaire contains 4 items, and focuses on individuals’ subjective thoughts and feelings related to perceived stressful experiences [42]. Respondents are asked to rate the statements on a five-point Likert-scale, ranging from 0 to 4, with two items being reverse-coded. The Hungarian version was translated by Stauder and Konkoly-Thege [47]. The internal reliability of the overall scale was found to be good in our sample (=0.85, ω = 0.85).

#### Young Schema Questionnaire-Short Form

The Young Schema Questionnaire-Short Form (YSQ-SF) was derived from the original, 205-item long Young Schema Questionnaire [43, 48]. The YSQ-SF contains 75 items, with 5 items for each of the 15 original scales. There are no reverse-coded items. The questionnaire was translated to Hungarian by Unoka, Tölgyes and Czobor [20]. The internal reliability was excellent for Emotional Deprivation (YSQ-ED) (=0.92, ω = 0.92), Abandonment/Instability (YSQ-Ab) (=0.91, ω = 0.91), Social Isolation (YSQ-SI) (=0.92 ω = 0.93), Defectiveness/Shame (=0.95, ω = 0.959j), good for Mistrust/Abuse (=0.88 ω = 0.88) and Unrelenting Standards (=0.87, ω = 0.87).

### Data Analysis

The data analysis was conducted using the computer software Jamovi version 2.5 [49]. The study focuses on two regression models. In both models, the independent variables are the same, while the two outcome variables differ (Healthy Orthorexia and Orthorexia Nervosa). The two models are combined in a single SEM (structural equation modeling) framework, so the relationship between the two outcome variables is not tested through separate correlations. In the SEM, the two orthorexia variables were the endogenous variables, while the covariates from the Young Schema Questionnaire served as exogenous variables. Perceived stress, age, BMI, and gender were also included as exogenous factors in the model. For the applied model, a minimum of 172 participants would be required according to GPower calculations, assuming a medium effect size (f² = 0.15), an alpha of 0.5, and a statistical power of 0.95.

In the following, we report the results, the effect sizes of the two models, and the effect of each variable (standardized beta) based on the SEM model. The SEM approach is particularly suitable for representing and interpreting the examined regression models within a single structure while simultaneously controlling for their mutual effects in a more concise manner. Regarding the effect of each variable, thresholds above 0.1 were considered low, above 0.3 medium, and above 0.5 high. In SEM, model fit is usually assessed through absolute and relative fit indices. However, in our model, all exogenous variables relate to all endogenous variables, creating a complete model where fit indicators would show perfect fit.

Before the analysis, the two regression models were run separately to test the assumptions. The purpose of testing these assumptions is to evaluate the model’s generalizability to the population. Multicollinearity was assessed by monitoring the VIF indicators. If the VIF indicators were below 10, multicollinearity could be ruled out. Additionally, homoscedasticity, the dispersion of residuals, and their normality were checked to assess how well they fit the regression line. To detect autocorrelation, the Durbin-Watson test was conducted to ensure there was no correlation among the error terms. Cook’s distance was used to identify and potentially exclude extreme cases that could distort the analysis.

Prior to constructing the model, we evaluated the assumptions of the regression analyses. The Shapiro-Wilk test and distribution plots showed a violation of normality for the two dependent variables (for more detailed descriptive statistics, see Table 4). However, there was no evidence of multicollinearity among the explanatory variables (VIF: 1.05–2.24), and no autocorrelation was detected. Additionally, no observations in the models had significant influence on the model estimates, based on Cook’s distance.

**Table 4:** Descriptive statistics of the variables (*n* = 1012)

	Mean	Median	SD	Minimum	Maximum	Shapiro-Wilk	
						W	*p*
Age	32.96	30.00	11.11	18.00	78.00	0.927	< 0.001
BMI	23.27	22.21	4.55	15.96	49.40	0.893	< 0.001
YSQ - ED	12.91	11.00	7.02	5.00	30.00	0.907	< 0.001
YSQ - Ab	12.83	11.00	6.82	5.00	30.00	0.907	< 0.001
YSQ - Mis	11.48	10.00	5.83	5.00	30.00	0.9	< 0.001
YSQ - SI	13.51	12.00	7.03	5.00	30.00	0.921	< 0.001
YSQ - Def	8.93	6.00	5.80	5.00	30.00	0.715	< 0.001
YSQ - US	17.60	18.00	6.43	5.00	30.00	0.98	< 0.001
TOS - HO	22.44	22.00	5.33	10.00	36.00	0.99	< 0.001
TOS - ON	11.79	11.00	3.94	8.00	31.00	0.841	< 0.001
PSS	13.45	14.00	3.35	4.00	20.00	0.982	< 0.001

*N* 1012, *YSQ – ED*, YSQ Emotional deprivation, *YSQ – Ab*, YSQ Abandonment/Instability, *YSQ – Mis*, YSQ Mistrust/Abuse, *YSQ – SI*, YSQ Social Isolation, *YSQ –Def*, YSQ Defectiveness/Shame, *YSQ –US*, YSQ Unrelenting standards, *TOS – HO*, TOS Healthy Orthorexia, *TOS –ON*, TOS Orthorexia nervosa, *PSS*, Perceived Stress Scale

When the variable TOS-ON served as the dependent variable, both heteroscedasticity and violation of the normal distribution of the residuals were observed. Consequently, we used a robust procedure (Unweighted Least Squares) to estimate the parameters of our combined model. Additionally, the confidence interval estimates of the parameters presented in the results table were adjusted through bootstrapping.

## Results

The results of the model showed that the explained variance ratio of the variance of TOS - HeOr was 11.7% (Wald χ2 [10] = 103.1, *p*<.001, R2 = 0.117), while for TOS - ON it was 11.5% (Wald χ2 [9] = 92.4, *p*<.001, R2 = 0.115). It can be seen that both models were significant. The correlation between the two variables was of medium effect size (*r*=.36, Z = 9.30, *p*<.001).

### Healthy orthorexia and early maladaptive schemas

YSQ-Emotional Deprivation, YSQ-Abandonment/Instability, YSQ-Defectiveness/Shame had no significant effect on TOS-HeOr. YSQ-Mistrust/Abuse ((β=−0.093, *p*=.037) had a negative association with TOS-HeOr, while YSQ-Social Isolation (β = 0.100, *p*=.042) had a positive effect on TOS-HeOr. In both cases, the effect size was weak. YSQ-Unrelenting Standards had the largest and also positive effect on TOS-HeOr (β = 0.195, *p*<.001).

### Orthorexia nervosa tendencies and early maladaptive schemas

**Fig. 1:**
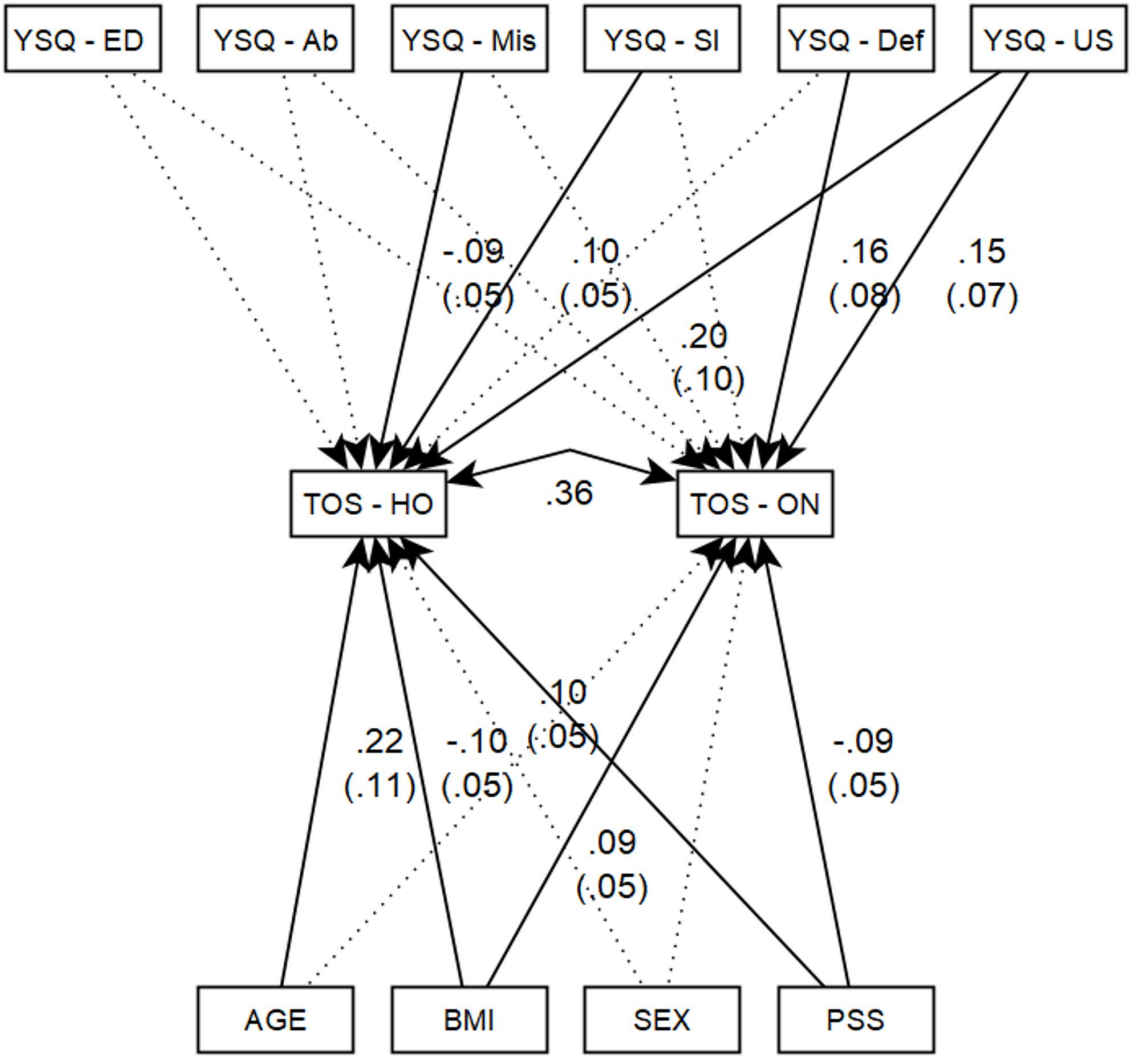
Structural equation model of orthorexia nervosa, healthy orthorexia and early maladaptive schemas. *YSQ – ED*, YSQ Emotional deprivation, *YSQ – Ab*, YSQ Abandonment/Instability, *YSQ – Mis*, YSQ Mistrust/Abuse, *YSQ – SI*, YSQ Social Isolation, *YSQ –Def*, YSQ Defectiveness/Shame, *YSQ –US*, YSQ Unrelenting standards, *TOS – HO*, TOS Healthy Orthorexia, *TOS –ON*, TOS Orthorexia nervosa. The standard error for the standardized coefficient is shown in brackets. The dotted lines indicate non-significant regression paths; in these cases, effect sizes and standard errors are not reported

TOS-ON only had significant associations with YSQ-Defectiveness/Shame (β = 0.163, *p*=.006) and YSQ-Unrelenting Standards (β = 0.145, *p*<.001). The other variables related to early maladaptive schemas had no significant effect on TOS-ON (Fig. 1; Table 5).

**Table 5:** Structual equation model of orthorexia nervosa, healthy orthorexia and early maladaptive schemas

Parameter Estimates				95% Confidence Intervals				
Dependent	Predictor	Estimate	SE	Lower	Upper	β	z	p
TOS - HeOr	YSQ - ED	−0.018	0.033	−0.078	0.049	−0.023	−0.545	0.586
	YSQ - Ab	−0.047	0.036	−0.120	0.030	−0.061	−1.308	0.191
	YSQ - Mis	−0.085	0.041	−0.163	0.001	−0.093	−2.082	0.037
	YSQ - SI	0.076	0.037	−5.57e − 4	0.148	0.100	2.034	0.042
	YSQ - Def	−0.085	0.045	−0.176	0.004	−0.092	−1.867	0.062
	YSQ - US	0.162	0.030	0.098	0.219	0.195	5.360	< 0.001
	PSS	0.166	0.065	0.042	0.303	0.104	2.566	0.010
	Age	0.106	0.018	0.071	0.142	0.220	5.916	< 0.001
	BMI	−0.111	0.041	−0.192	−0.032	−0.095	−2.723	0.006
	Sex	0.402	0.618	−0.763	1.632	0.021	0.651	0.515
TOS - ON	YSQ - ED	−0.041	0.024	−0.085	0.010	−0.073	−1.696	0.090
	YSQ - Ab	0.012	0.030	−0.046	0.069	0.021	0.394	0.694
	YSQ - Mis	0.052	0.033	−0.014	0.117	0.076	1.560	0.119
	YSQ - SI	−0.002	0.026	−0.052	0.051	−0.003	−0.070	0.944
	YSQ - Def	0.111	0.041	0.029	0.189	0.163	2.739	0.006
	YSQ - US	0.089	0.022	0.048	0.136	0.145	4.065	< 0.001
	PSS	−0.109	0.048	−0.197	−0.011	−0.092	−2.253	0.024
	Age	0.007	0.013	−0.020	0.033	0.020	0.550	0.583
	BMI	0.080	0.033	0.010	0.141	0.092	2.426	0.015
	Sex	0.285	0.539	−0.761	1.463	0.020	0.528	0.597

*YSQ – ED*, YSQ Emotional deprivation, *YSQ – Ab*, YSQ Abandonment/Instability, *YSQ – Mis*, YSQ Mistrust/Abuse, *YSQ – SI*, YSQ Social Isolation, *YSQ –Def*, YSQ Defectiveness/Shame, *YSQ –US*, YSQ Unrelenting standards, *TOS – HO*, TOS Healthy Orthorexia, *TOS –ON*, TOS Orthorexia nervosa, *PSS*, Perceived Stress Scale

### Orthorexia nervosa tendencies, healthy orthorexia, demographical variables, BMI and stress

Age had a significant effect on TOS-HeOr (β = 0.220, *p*<.001) but had no effect on TOS-ON (β = 0.020, *p*=.583). Sex had no significant effect on either of the TOS variables (β = 0.021, *p*=.515; β = 0.020, *p*=.597). BMI had a significant and negative effect on TOS-HeOr (β=−0.095, *p*=.006), and a significant and positive effect on TOS-ON (β = 0.092, *p*=.015), and the effect size was weak in both cases. TOS-HeOr had a weak positive association with PSS (β = 0.104, *p*=.010), while PSS had a weak negative effect on TOS-ON (β=−0.091, *p*=.024). For the complete model, see Fig. 1; Table 5.

## Discussion

Our goal was to examine and compare maladaptive schemas between individuals who maintain healthy eating habits and those with ON tendencies. Since the connection between ON and early maladaptive schemas has not yet been studied, our research focused on the relationship between EMS and other eating disorders. According to our analyses, HeOr showed a positive correlation with the social isolation and unrelenting standards schemas, while the SEM model indicated a positive relationship between ON and the unrelenting standards and defectiveness/shame schemas.

### Orthorexia nervosa tendencies, healthy eating, BMI and perceived stress

The results showed a positive association between ON tendencies and BMI, while the relationship between HeOr and BMI was negative. Previous research has reported inconsistent findings regarding ON tendencies and BMI, although some studies have indicated a positive correlation [50]. One possible explanation may be that individuals with higher BMI or those who are overweight may be more likely to developing ON tendencies, possibly as a response to weight-related discrimination or body dissatisfaction, which can lead to increased dietary control [51, 52]. Additionally, research has documented cases of individuals with ON tendencies experiencing a loss of control over eating, which may further contribute to the relationship with BMI [16, 53, 54].

HeOr was positively associated with perceived stress, while ON tendencies showed a negative association with it. This result, which might seem surprising at first, can be understood in several ways. First, the observed links may reflect the self-regulating role of ON tendencies. Past research has identified affect regulation as the second most common motivation for food choices among people with ON tendencies [4, 55]. This aligns with findings on other eating disorders: restriction or purging are often used by individuals with eating disorders to cope with emotional distress [56, 57]. The negative association between ON tendencies and perceived stress may also relate to the ego-syntonic nature of the disorder. People with ON tendencies often fail to recognize the harmful effects of their habits (e.g. weight loss, fatigue, or anxiety) [58].

Additionally, creating new dietary rules and predicting their results can evoke positive emotions, such as excitement [53, 58]. A 2021 study with elderly individuals reported similar results: ON tendencies were positively linked to body appreciation and life satisfaction, with no significant connection to psychological distress or eating disorder symptoms [59]. One possible explanation for this unexpected pattern is that the questionnaire used in the study may have involved conceptual overlap between ON tendencies and healthy eating. If orthorexic behaviors are not clearly distinguished from normal health-conscious habits, the measurement might partly reflect adaptive tendencies, which could obscure their relationship with stress. When differentiating ON tendencies from healthy eating, it’s important to consider factors, such as age and cultural background. Additionally, assessment tools that separately measure functional impairment and emotional distress, beyond general health behaviors, may offer a clearer understanding of ON tendencies.

### Orthorexia nervosa tendencies, healthy eating and early maladaptive schemas

Perfectionism and unrelenting standards have been connected to the development and maintenance of eating disorders, such as AN and bulimia nervosa [20, 21, 60–62]. Our findings suggest that stronger activation of the unrelenting standards schema may also contribute to the development and persistence of ON. This schema can influence adherence to strict dietary rules, the pursuit of perfectly clean eating and striving for optimal health, and may also lead to a critical attitude toward those who do not follow a healthy diet [14, 58]. Activation of this schema may cause individuals to judge themselves harshly and engage in self-punishment if they deviate from their strict diet [62, 63]. By following rigid rules linked to unrelenting standards, individuals may also experience an illusion of control, which has been shown to be an important factor in ON tendencies [58, 63].

The unrelenting standards schema showed a stronger association with healthy eating than with ON tendencies. Although the difference between the two effect sizes is narrowly significant, it does not indicate a substantial difference (Z = 1.99, *p* =.047). Activation of this schema may be relevant in both healthy eating and ON tendencies. This is not surprising, as adhering to rules is necessary for maintaining a healthy diet. The similar levels of schema activation in ON tendencies and healthy eating may have several explanations. First, individuals with high levels of activation of this schema often do not see their standards as unrealistic [14]. As a result, in self-report questionnaires, they may underestimate the extent of this schema and report lower ratings. Additionally, ON tendencies are usually ego-syntonic, meaning that affected individuals tend to underestimate or deny the presence and severity of their problems, which can further distort self-report outcomes [58, 64].

The model demonstrated a positive association between the defectiveness/shame schema and ON tendencies. Several studies have connected low self-esteem to ON tendencies, and we propose that for individuals with ON tendencies, the pursuit of extreme dietary control might help reinforce their self-esteem [8, 23, 30, 33, 65, 66]. The sense of superiority seen in individuals with ON tendencies supports this idea [1, 17, 63]. According to schema theory, overcompensation is a coping strategy in which individuals act as if the opposite of their schema is true [14]. In the context of the defectiveness/shame schema, overcompensation often manifests as perfectionism, arrogance, or critical and devaluing attitudes toward others [14]. By striving for dietary perfection, viewing themselves as more disciplined or pure, and making downward social comparisons, individuals with ON tendencies may try to counteract feelings of defectiveness and elevate their self-worth [62].

The social isolation schema demonstrated a significant association with HeOr, but not with ON. This may be because individuals with ON tendencies often surround themselves with others who share similar beliefs and habits [67]. Additionally, their lives often revolve so heavily around eating that the need for social connection becomes less important [2, 68]. Past research also suggests personality differences between ON and HeoR; for example, HeOr has been associated with psychoticism, characterized by greater distrust and suspicion, potentially leading to self-isolation [69]. It is also possible that healthy eating habits themselves are influenced by the social isolation schema, as individuals unconsciously engage in actions that reinforce their maladaptive schemas [14]. The characteristics of the questionnaire may also help explain these results. Previous studies have found correlations ranging from 0.31 to 0.74 between the HeOr and ON scales; in our sample, the correlation was 0.36 [7, 10, 45, 70]. This indicates some overlap between healthy eating and ON tendencies, likely because behavioral aspects of healthy eating are shared by both groups. The HeOr scale mainly measures these behaviors, while differences appear on the ON scale, which includes items assessing emotional distress related to healthy eating (e.g., worry, guilt) [7]. Moreover, individuals who eat healthily and score high on the HeOr scale may be manifesting mild ON tendencies or may lack awareness of the distress and functional impairment arising from their eating behaviors [58, 62, 68].

This emphasizes the need for future studies that explore different dimensions (e.g. emotional distress, behaviors, and impairments across various life areas) separately, rather than viewing orthorexia nervosa tendencies as a single dimension. One limitation of the TOS may be that it does not differentiate between the various aspects of ON tendencies, thereby highlighting the necessity for future research to focus on this. Additionally, the YSQ measures functioning subordinate to schemas, which may also affect the results. People with eating disorders often engage in schema avoidance, including primary (schema overcompensation) and secondary avoidance (affect reduction after schema activation), which may hide or distort the activation of relevant schemas in self-report data [16, 71, 72].

### Limitations

Due to the cross-sectional design of this study, it is not possible to draw conclusions regarding causality. Furthermore, the use of convenience sampling and a nonrepresentative sample limits how much the findings can be generalized. It should also be acknowledged that the majority of the questionnaires were distributed to individuals with a preexisting interest in healthy eating, suggesting that future studies should include more diverse populations in order to provide a more comprehensive understanding, In addition, nearly half of the respondents had to be excluded, either because they did not complete the full questionnaire battery or due to concerns about data quality. While our analyses indicated that our screening process did not introduce bias into the sample, the high rate of incomplete responses raises the question of why dropout rates were so substantial. This is an issue that warrants further consideration in future research, particularly to find ways to reduce dropout rates.

Moreover, orthorexia nervosa still cannot be considered an official diagnosis. One of the reasons for this is the ongoing challenges of conceptualization, given its similarities to other disorders [5, 17]. Our findings also support this view and suggest that ON tendencies might be better understood along a continuum, using a dimensional approach in future research.

Another limitation of the research is that BMI values were based on self-report, which may have led to inaccuracies in the classification of BMI categories. While the overall sample size was adequate, there were notable imbalances in gender representation and other demographic characteristics, such as educational level. Future studies should strive for a more balanced inclusion of male participants and a broader range of educational backgrounds to enhance generalizability. Although participants were asked to declare that they had not receiveed treatment for, or experienced, neurological or psychiatric disorders, this criterion does not account for individuals who may have been unaware of such conditions. Furthermore, the structural equation modeling revealed only weak effect sizes for significant correlations. The study also used the shorter version of the YSQ and assessed only six early maladaptive schemas.

Given the preliminary nature of this study and the lack of prior research focusing on the association between ON tendencies and EMS, it is recommended that future investigationss assess all 18 early maladaptive schemas. Additionally, there are few studies that have explored group-level patterns in ON tendencies. Using cluster analysis may facilitate the identification of distinct ON tendency profiles and their associations with EMS or other relevant constructs, such as perfectionism or health anxiety. Lastly, qualitative studies could provide valuable insights into the interplay between early maladaptive schemas and ON tendencies. Conducting in-depth interviews may help explore the development and maintenance of specific schemas in individuals exhibiting ON tendencies. Future research could also consider the roles of family dynamics and cultural influences in the development of ON tendencies.

## Conclusions

No previous study has examined the relationship between orthorexia nervosa tendencies and early maladaptive schemas. However, our research confirms that this area is worth further investigation. The results suggest a potential association with schemas related to self-esteem and perfectionism. Investigating early maladaptive schemas may bring us closer to understanding the nature and development of orthorexia nervosa tendencies and the challenges the affected individuals may face.

## Data Availability

The datasets used and/or analyzed during the current study are available from the corresponding author on reasonable request.

## Abbreviations

EMS: Early maladaptive schemas
ON: Orthorexia nervosa
OCD: Obsessive-compulsive disorder
OCPD: Obsessive-compulsive personality disorder
AN: Anorexia nervosa
TOS: Teruel Orthorexia Scale
YSQ-SF: Young Schema Questionnaire Short-Form
HeOr: Healthy orthorexia
YSQ-ED: Emotional deprivation
YSQ-Ab: Abandonment/instability
YSQ-Mis: Mistrust/abuse
YSQ-SI: Social isolation
YSQ-Def: Defectiveness/shame
YSQ-US: Unrelenting standards
PSS: Perceived Stress Scale
